# Crisis communication strategies for health officials

**DOI:** 10.3389/fpubh.2022.796572

**Published:** 2022-11-22

**Authors:** Zhaohui Su, Huan Zhang, Dean McDonnell, Junaid Ahmad, Ali Cheshmehzangi, Changrong Yuan

**Affiliations:** ^1^School of Public Health, Southeast University, Nanjing, China; ^2^School of Social Development and Public Policy, Beijing Normal University, Beijing, China; ^3^Department of Humanities, South East Technological University, Carlow, Ireland; ^4^Rufaidah Nursing College, Peshawar, Pakistan; ^5^Faculty of Science and Engineering, University of Nottingham Ningbo China, Ningbo, China; ^6^Network for Education and Research on Peace and Sustainability (NERPS), Hiroshima University, Hiroshima, Japan; ^7^School of Nursing, Fudan University, Shanghai, China

**Keywords:** crisis communication, health officials, COVID-19, public health, health communication

## Abstract

**Background:**

Mixed messaging among health officials are prevalent amid COVID-19. Crisis communication strategies have the potential to help health officials effectively address issues such as mixed messages and improve their crisis communication efficacy. However, there is a dearth of insights in the literature. Therefore, to bridge the research gap, this study aims to examine practical strategies health officials can utilize to improve their crisis communication efficacy.

**Methods:**

A literature review on effective crisis communication strategies amid COVID-19 was conducted in PubMed, Scopus, and PsycINFO, with a focus on scholarly literature published in English.

**Results:**

The findings of the study identified the following strategies that health officials can utilize to improve their crisis communication capabilities: (1) develop fact-based, transparent, and accountable messaging, (2) utilize people-centered and empathetic persuasive strategies, and (3) leverage international collaboration for consistent messaging and comprehensive crisis communication.

**Conclusion:**

COVID-19 has challenged health officials with unprecedented crisis communication duties and responsibilities. In this study, we underscored the importance of effective crisis communication amid global health emergencies like COVID-19, and identified communication strategies health officials could adopt or adapt to improve their crisis communication efficacy. Future research could explore strategies health officials can use to better communicate with government officials and media professionals to further help health officials improve their crisis communication capabilities, their abilities to avoid preventable miscommunication or mixed messaging, and in turn, society's collective strengthen in curbing and controlling the pandemic.

## Background

Crises are ubiquitous in healthcare (1). Ranging from everyday medical disputes (e.g., medical violence), periodical epidemics (e.g., seasonal influenza outbreaks), to once-in-a-century global pandemics (e.g., coronavirus disease 2019 or COVID-19), health officials often have to cope with emergency events on a daily basis (2–5). Take the COVID-19 pandemic for instance. As of mid-April, 2022, global COVID-19 cases has surpassed 500 million, while total deaths reached over 6 million (6). Accumulated evidence suggests that not only the pandemic is unprecedented, it evolves fast, as seen in the escalation of the transmissions of the Delta, Omicron, and then the BA.2 subvariant across the globe (7). This, in turn, may have partially contributed to the poor crisis communication practices among health officials across the pandemic (8, 9). For instance, three of the arguably most influential health officials in the U.S., the director of CDC Dr. Robert Redfield, the U.S. Surgeon General Jerome Adams, and the director of the National Institute of Allergy and Infectious Diseases Dr. Anthony Fauci, all have wrongly dismissed face masks' critical role in preventing COVID-19, in public, on record, and often on multiple occasions (10).

Dr. Fauci, for instance, said on record in a television interview that was directed to the general public “there's no reason to be walking around with a mask,” while addressing the role of masking amid COVID-19 (10). Many thanks to the ever-presence COVID-19 infodemics, the statement was paraphrased into “masks are not good”, and subsequently referenced a sobering number of times by various public figures, social media influencers, media outlets, and perhaps most alarmingly, conspiracy theorists (10–12). It is important to note that these three public health figures are only representatives of the pool of health officials that have issued and popularized mixed messages that range from confusing to conflicting (13–19). Accumulating evidence shows that health officials, including those working at the World Health Organization (WHO), arguably the most authoritative organization in healthcare directives, often fall victim to poor crisis communication practices that have resulted in ineffective pandemic communication, ranging from mixed narratives, conflicting advice, to poor communication skills (e.g., self-contradictory and confusing guidelines for masking) (20–24).

Considering that the pandemic is still evolving, it might be difficult to pinpoint the exact human and economic consequences of these contradictory statements (25–27). What is clear, though, is that failing to communicate with the public effectively about COVID-19 imperatives can cause substantial confusion in the public and negatively impact people's compliance with safety measures (28, 29). In addition, inconsistent health directives could also deteriorate people's trust and confidence in health officials and the government at large (30, 31). Not to mention that contradictory statements can ignite criticism from the public and demand additional communication efforts to further elaborate the messages, which in turn, could increase health officials' workload and fuel the physical and mental burnout many of them face constantly (32).

One way to address this issue is *via* effective crisis communication. Crisis communication could be understood as health officials' abilities to effectively, efficiently, and empathetically communicate and collaborate with key stakeholders in times of crisis, with the ultimate goal of controlling and containing emergency events and in turn, protecting personal and public health. Crisis communication, when coupled with persuasive strategies, has the potential to help health officials address issues such as mixed messages and improve their communication efficacy (33–43). However, though urgent attention is needed to address health officials' communication efficacy amid COVID-19, there is a dearth of research available in the literature (44). Therefore, to bridge the research gap, this study aims to examine practical strategies health officials can utilize to improve their crisis communication efficacy.

## Methods

A review of the literature published in the COVID-19 context was conducted in PubMed, Scopus, and PsycINFO on December 12, 2021. Search terms used were: (“crisis communication strateg^*^”[Title/Abstract] OR “crisis communication method^*^”[Title/Abstract] OR “ crisis communication mechanism^*^”[Title/Abstract] OR “ crisis communication practice^*^”[Title/Abstract] OR “ crisis communication intervention^*^”[Title/Abstract]) AND (“covid 19”[Title/Abstract] OR “covid-19”[Title/Abstract] OR “SARS-CoV-2”[Title/Abstract] OR “2019-nCoV”[Title/Abstract] OR “novel coronavirus”[Title/Abstract] OR “new coronavirus”[Title/Abstract] OR “coronavirus”[Title/Abstract]). Key information on crisis communication strategies amid COVID-19 was obtained. Table 1 lists the selection criteria adopted in screening the articles. Overall, studies were excluded if they: (1) did not focus on COVID-19 [e.g., foods-related crises (45)], (2) did not offer insights on crisis communication from health officials' perspectives [e.g., articles focused on government officials (46)], (3) did not discuss or identify crisis communication strategies, and (4) were not written in English.

**Table 1:** Study inclusion criteria.

**Category**	**Criteria**
Study context	COVID-19
Communication context	Crisis communication (as opposed to risk communication)
Language	English
Research focus	Crisis communication strategies for health officials amid COVID-19
Study type	Empirical and non-empirical research
Study outcome	Effective crisis communication strategies

## Results

**Figure 1:**
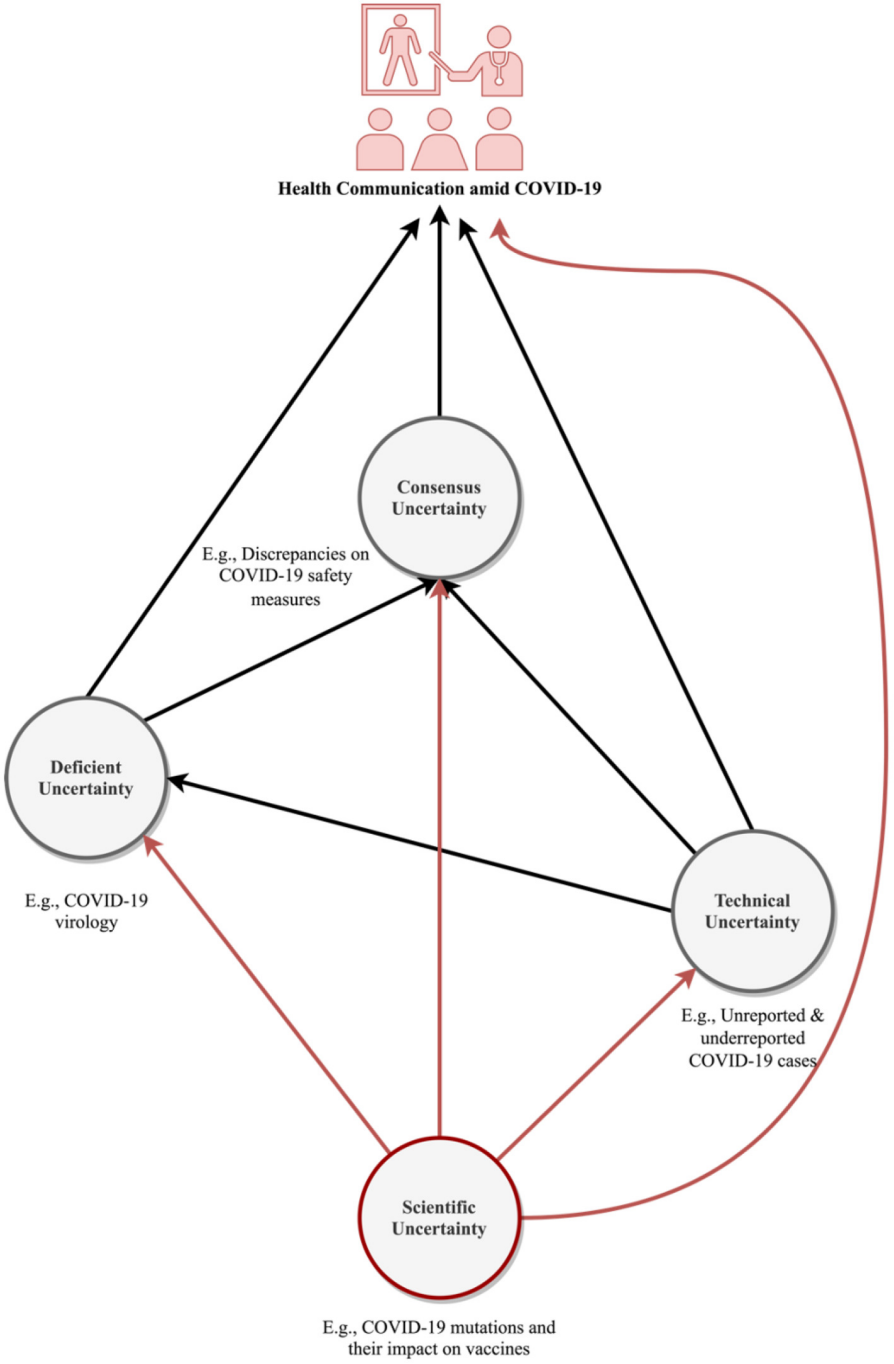
A schematic representation of the interplay between COVID-19 communication and uncertainties surrounding the pandemic.

The search yielded 107 records. After the reviewing process, 18 peer-reviewed papers met the eligibility criteria and were subsequently included in the final review (see Table 2). The results indicate that, in addition to (1) a lack of data and evidence (“known unknowns,” what scholars refer to as the deficient uncertainty), (2) measurement errors, such as unreported and underreported COVID-19 cases (technical uncertainty), and (3) a lack of consensus about COVID-19 and best approaches to control it (consensus uncertainty), the ever-evolving nature of COVID-19 (e.g., virus mutations), may further result in (4) scientific uncertainty about the pandemic (47), which could result in health officials' poor messaging amid COVID-19, and subsequently, contribute to their suboptimal crisis communication capabilities (Figure 1).

**Table 2:** List of included articles.

**Author**	**Year**	**Title**
Drescher et al. (48 )	2021	The spread of COVID-19 crisis communication on Twitter: The effect of structure, content and style of COVID-19 tweets of German public authorities and experts
Ece (49)	2022	Health Communication Strategies: Crisis Management and Infodemic During COVID-19
Jong (50)	2020	Evaluating crisis communication. A 30-item checklist for assessing performance during COVID-19 and other pandemics
Kwok et al. (51)	2021	Crisis communication on social media: what types of COVID-19 messages get the attention?
MacKay et al. (52)	2021	Examining social media crisis communication during early COVID-19 from public health and news media for quality, content, and corresponding public sentiment
Ngai et al. (53)	2020	Grappling with the COVID-19 health crisis: content analysis of communication strategies and their effects on public engagement on social media
Noar et al. (54)	2020	(Mis)communicating about COVID-19: Insights from health and crisis communication
Paek et al. (55)	2021	Information Communication Technologies (ICTs), crisis communication principles and the covid-19 response in South Korea
Pang (56)	2021	Leadership and crisis communication during COVID-19: The case of Brunei Darussalam
Radanović Felberg (57)	2021	“Norwegian-Somalis are best suited to inform Norwegian-Somalis”: Crisis communication, linguistic diversity and social (in)equality during the initial stages of the Covid-19 pandemic as represented by the Norwegian Broadcasting Corporation (NRK)
Ratzan et al. (58)	2020	Enhancing global health communication during a crisis: lessons from the COVID-19 pandemic
Shulman et al. (59)	2020	Don't dumb it down: The effects of jargon in COVID-19 crisis communication
Shulman et al. (60)	2021	The interplay of jargon, motivation, and fatigue while processing COVID-19 crisis communication over time
Su et al. (61)	2021	Mental health consequences of COVID-19 media coverage: the need for effective crisis communication practices
Subert (62)	2021	A gender-sensitive approach to U.S. crisis communication for COVID-19 and beyond
Tetteh (63)	2020	A leader's guide to crisis communication: lessons from Ebola for COVID-19
Wagner et al. (64)	2021	“The part played by people” in times of COVID-19: interpersonal communication about media coverage in a pandemic crisis
Wu et al. (65)	2020	COVID-19: peer support and crisis communication strategies to promote institutional resilience

The findings of the study identified the following strategies that health officials can utilize to countermeasure the abovementioned compounding factors, and in turn, improve their crisis communication capabilities: (1) develop fact-based, transparent, and accountable messaging, (2) utilize people-centered and empathetic persuasive strategies, and (3) leverage international collaboration for consistent messaging and comprehensive communication (48–65). These strategies will be discussed in detail in the following sections.

## Discussion

**Figure 2:**
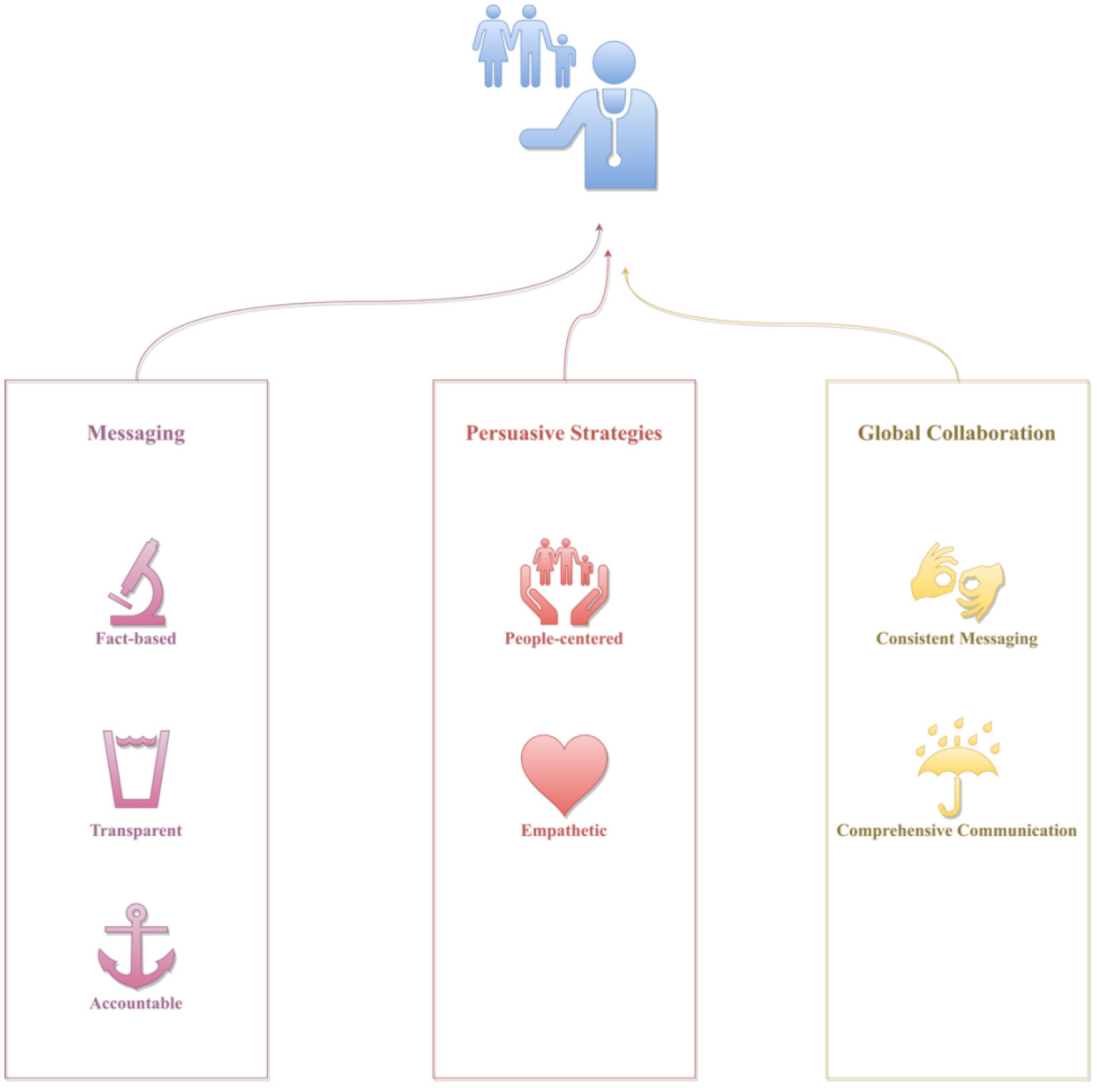
A schematic representation of the identified crisis communication strategies.

This study set out to examine practical strategies health officials can utilize to improve their crisis communication efficacy. This study is among the firsts that examined actionable strategies health officials can adopt or adapt to improve their COVID-19 communication efficacy. The results of the study suggest that developing fact-based, transparent, and accountable messaging, incorporating people-centered and empathetic persuasive strategies, and leveraging international collaboration for consistent messaging and comprehensive communication can help health officials better manage crisis communication amid COVID-19 more effectively. A schematic representation of these strategies could be found in Figure 2. Details of these strategies will be discussed in the following sections.

### Effective crisis communication strategies

#### Fact-based, transparent, and accountable messaging

A key effective crisis communication strategy is to develop fact-based, transparent, and accountable messaging (66–71). It is of critical importance that health officials base their statements on scientific facts, and communicate the key messages clearly and consistently with the public, including important caveats if the evidence shared was preliminary and subject to imminent change. Instead of merely emphasizing the core health message, health experts also should underscore limitations to the current knowledge base upon which the message is developed—that the message is derived “based on latest evidence” or “according to what we know so far.” This approach will not only make sure health officials are responsibly communicating the facts and directives they ask the public to believe and follow, but also build rapport between health officials and the public.

Research on 6,000 Americans shows that while downplaying the uncertainty of COVID-19 can elicit support from the audience in the short term, reversals in projections can substantially reduce the message sender's scientific merit (72). Findings on 2,011 people living in Germany also show that most of the respondents prefer open discussion about COVID-19 uncertainties (73). These insights, overall, suggest that ignoring or downplaying uncertainties could harm health experts' credibility among members of the public, and further underscores the importance of transparent and accountable communication. Take the Omicron variant for instance. While it is critical that health experts support their crisis communication with facts, it is equally important, if not more, for them to communicate transparently and accountably—making sure the public understands that the current “knowns” about Omicron are in flux, and that scientists worldwide are working nonstop to unravel the “unknowns” about the variant to keep the public informed. It is important to underscore that it should be up to the public to decide if the style or substance of the communication should be “dumbed down” (59), rather than public health officials.

To be honest about what is known and what is subject to change about the pandemic, health officials are effectively making their messages more relevant and relatable to the public. Overall, many approaches can help health officials to clearly and responsibly communicate COVID-19 messages with the public, such as using visuals to accompany the message (e.g., interactive videos), adopting different narrative frames (e.g., promotion-focused vs. prevention-focused), and incorporating varied language formats (34–39). For instance, rather than framing health messages as hard truth, health experts can use clear and relatable language to explain the intricacies of health communication amid COVID-19, such as “Health mandates and policies amid COVID-19 are like software—for our benefits, they have to be updated, as their abilities to address public health imperatives get better with each update.” This “full disclosure” step is essential, as once the public understands what to expect and why they will have the opportunity to adjust their mindset and are less likely to distrust or lose confidence in health officials and governments in general.

#### People-centered and empathetic persuasive strategies

People-centered crisis communication requires health officials to prioritize people's interests over politics and profits, whereas empathetic crisis communication needs health officials to factor in key contextual factors, such as the emotional burden and physical burnout the public might have already been shouldering throughout the pandemic (61), while delivering the essential pandemic updates. As of December 20, 2021, COVID-19 has already caused 275 million infections and 5.35 million deaths worldwide (74), along with its sobering impacts on people's mental health (75). In light of the ever-growing toll on lives, livelihoods, and economies that COVID-19 has exerted on the public, people-centered communication requires health experts to not only communicate fact-based, transparent, and accountable messages, but also convey care and empathy to the public as well (76).

In other words, health experts should make COVID-19 communication personable and relatable (77), and when possible, address the public's cognitive (e.g., information about COVID-19 vaccines), affective (e.g., fear and stress associated with receiving or not receiving a COVID-19 vaccine), and behavioral needs (e.g., lack of motivation or capabilities to uptake a COVID-19 vaccine) (42, 62, 78, 79). It is important to note that having a deep and comprehensive understanding of the target audience's characteristics is essential to effective communication (38, 42, 43), as it is not only essential to yielding desired health behavioral outcomes in the public, but also important to avoid potential unintended consequences that could harm individuals' mental health and wellbeing [e.g., anxiety (80); racism or stigmatization (81)].

For instance, one of the recurring reasons for African Americans' distrust in vaccines can be traced back to the Tuskegee Syphilis Study (82)— health and government officials deliberately denied African American patients' medicine that can effectively treat syphilis, just to observe and collect data about the disease's progression (83). In light of these insights and according to ELM, to effectively communicate the importance of COVID-19 vaccines to personal and public health with African Americans, rather than emphasizing vaccine efficacy statistics that African Americans may distrust, health officials should consider collaborating with already trusted figures in the community, such as African American healthcare professionals and social media influencers, to stimulate conversations about adopting COVID-19 vaccines (84–87).

One good example is the selection of Sandra Lindsay, an African American nurse working at the Long Island Jewish Medical Center in New York City, as the first person who received a COVID-19 vaccine in the U.S. (88). Leveraging this high profile and heavily mediated event, the symbolic meaning of this communication endeavor is threefold: (1) to send a message to the public that COVID-19 vaccines are safe to take, (2) to encourage African Americans across the country to update COVID-19 vaccines, and possibly (3) to persuade the Jewish community in New York city to uptake the vaccine as well, a community which has been defiant in responding to government's COVID-19 safety measures (89). Overall, it is important to underscore that the cornerstone of crisis communication is the people—how to communicate effectively amid crises so that the public and the health officials can build back a new normal speedily and successfully. In other words, crisis communication should not merely focus on disseminating facts and figures; it should be centering on utilizing tailored people-centered and empathetic persuasive strategies to leverage factual messages to maximize their potential to inform, and engage, and empower the public to better cope with the crises.

#### International collaboration for consistent and comprehensive communication

As trusted public figures, health officials across the world have a fiduciary duty to the public to find the best possible solution in controlling COVID-19. One of the most cost-effective ways to accomplish this objective is via pooling scientific expertise and unifying COVID-19 communication strategies from international health officials, as international cooperation and collaboration can help: (1) bridge potential gaps in different governments' COVID-19 communication strategies, (2) broaden our collective understanding of effective ways to communicate about COVID-19, (4) improve the public's compliance with COVID-19 safety measures, (3) better equip global health systems for future pandemics (90). A key consideration is that individual nations could often fail to provide comprehensive or complete knowledge or know-how on COVID-19 single-handedly (52).

When the “there's no reason to be walking around with a mask” statement was made by Dr. Fauci on March 8^*th*^, 2020, almost two months after China shared the very first COVID-19's genetic sequence with the World Health Organization (WHO) (January 11^*th*^, 2020), evidence was available on the effectiveness of COVID-19 safety measures in many countries across the world (66–71). Take China for instance. On December 31^*st*^, 2012, 27 cases of pneumonia of unknown causes were reported in Wuhan. Less than a month later (January 23^*rd*^, 2020), the city of Wuhan initiated its lockdown—the single largest quarantine in recorded history (16). In February, 2020, China has opened its first Fangcang hospital that has the ability to hold 13,000 beds, with 13 more of these hospitals under construction. Yet by March 10^*th*^, 2020, these Fangcang hospitals were no longer needed.

In October, 2020, data showed that China's economy is the first to bounce back amid the pandemic—it is projected to be the only world's major economy to: (1) report a positive gain at year-end and (2) have an up to 9% GDP growth in 2021 (91). One key reason for China's successful management of COVID-19 centers on its effective crisis communication—against all odds, health officials have managed to persuade most of its 1.4 billion people to comply with COVID-19 safety measures such as masking, maintaining personal hygiene, and social distancing (92–95). Overall, effective communication practices can be found in many countries across the world, ranging from Finland, Ireland, New Zealand, Senegal, South Korea, to Vietnam (66–71, 96–99).

Take another nation, Senegal, for instance. Though it only has seven doctors for every 100,000 people, many thanks to its health and government officials' clear, consistent, and science-based communication about COVID-19 and what actions the government and its citizens need to be taken to control the pandemic (100), with a 16 million population, Senegal only have approximately 17,758 infections and 365 cases as of December, 2020 (101). These insights, overall, underscore the crucial imperative for international collaboration in thwarting COVID-19 (102). COVID-19 is a global health crisis—if the virus can cross borders and scientists across the globe can work together to develop COVID-19 vaccines, surely health officials worldwide can work collectively and collaboratively, above and beyond their political or ideological differences, to leverage international collaboration to develop more updated and collaborated crisis communication strategies and COVID-19 messages to better cope with the pandemic.

COVID-19 is also unprecedented, and to effectively control the pandemic, we need unprecedented levels of international cooperation and collaboration that bypass or transcend geopolitical concerns or “pandemic nationalism.” While fighting infectious diseases can be accomplished by individual countries, cost-effectively controlling a pandemic of COVID-19's scale, both in terms of macro-level evidence-based decision-making and micro-level empathetic and effective interventions, requires health experts across the globe to work together and collaboratively (103–106). Overall, communication strategies—fact-based, transparent, and accountable communication, coupled with people-centered and empathetic persuasive strategies, developed based on international cooperation and collaboration, can help health officials across the globe manage COVID-19 more effectively, and get a head start in preparing for future health crises (107).

## Limitations

While this study fills critical gaps in the literature, it is not without limitations. First, this study is not a systematic review, which means that the results of this study are limited in reproductivity and replicability. We excluded articles that focused on government officials or politicians' crisis communication practices. This means that studies that categorize health officials as government officials were not included in the review. Furthermore, only scholarly literature in English was reviewed and analyzed in the study, which suggests that it is possible that potential useful insights from COVID-19 articles in non-English language or non-academic in nature were not represented in the current study. To address these limitations, future research could adopt a systematic review approach that covers multiple languages to further shed light on COVID-19 crisis communication strategies for health officials.

## Conclusion

COVID-19 has challenged health officials with unprecedented crisis communication duties and responsibilities. In this study, we underscored the importance of effective crisis communication amid global health emergencies like COVID-19, and identified communication strategies health officials could adopt or adapt to improve their crisis communication efficacy. Future research could explore strategies health officials can use to better communicate with government officials and media professionals to further help health officials improve their crisis communication capabilities, their abilities to avoid preventable miscommunication or mixed messaging, and in turn, society's collective strengthen in curbing and controlling the pandemic.

## Data availability statement

The raw data supporting the conclusions of this article will be made available by the authors, without undue reservation.

## Author contributions

ZS conceived the work, reviewed the literature, drafted, and edited the manuscript. HZ, DM, JA, AC, and CY reviewed the literature and edited the manuscript. All authors approved the manuscript for submission.

## Funding

This work was supported by the Fundamental Research Funds for the Central Universities.

## Conflict of interest

The authors declare that the research was conducted in the absence of any commercial or financial relationships that could be construed as a potential conflict of interest.

## Publisher's note

All claims expressed in this article are solely those of the authors and do not necessarily represent those of their affiliated organizations, or those of the publisher, the editors and the reviewers. Any product that may be evaluated in this article, or claim that may be made by its manufacturer, is not guaranteed or endorsed by the publisher.
